# Health in the New Scenarios for Climate Change Research

**DOI:** 10.3390/ijerph110100030

**Published:** 2013-12-19

**Authors:** Kristie L. Ebi

**Affiliations:** ClimAdapt, LLC, 424 Tyndall Street, Los Altos, CA 94022, USA; E-Mail: krisebi@essllc.org

**Keywords:** climate change, scenarios, health, adaptation, mitigation

## Abstract

The climate change research community is developing a toolkit for creating new scenarios to explore and evaluate the extensive uncertainties associated with future climate change and development pathways. Components of the toolkit include pathways for greenhouse gas emissions over this century and their associated magnitude and pattern of climate change; descriptions of a range of possible socioeconomic development pathways, including qualitative narratives and quantitative elements; and climate change policies to achieve specific levels of radiative forcing and levels of adaptive capacity. These components are combined within a matrix architecture to create a scenario. Five reference socioeconomic development pathways have been described along axes describing increasing socioeconomic and environmental challenges to adaptation and to mitigation. This paper extends these global pathways to describe their possible consequences for public health and health care, and considers the additional elements that could be added to increase the relevance of the new scenarios to address a wider range of policy relevant questions than previously possible.

## 1. Introduction

The future is inherently uncertain, calling for the use of scenarios to describe the range and character of possible futures. A *scenario* is a comprehensive and plausible description of the future of the human-environment system, including a narrative with qualitative trends and quantitative projections relevant to development patterns [1]. Scenarios facilitate exploring and evaluating the range and character of uncertainties associated with future climate change and development pathways. Scenario-based analyses are critical to furthering interdisciplinary analysis and assessment of the possible risks of climate change to physical, natural, and human systems; and furthering evaluation of the effectiveness of mitigation and adaptation options to avoid, prepare for, and manage those risks. Projecting possible impacts under different futures and identifying the trade-offs and synergies of adaptation and mitigation policies requires not just scenarios of greenhouse gas emissions and resulting climate change, but also descriptions of how future socioeconomic development pathways could increase or decrease the risks human and natural systems are likely to face under different climates.

Independent of climate change, socioeconomic factors will alter future burdens of climate-sensitive health outcomes and the status of public health and health care infrastructure. These, in turn, will interact with climate change, leading to different risks under different development pathways. Many projections of the health risks of climate change make limited to no explicit socioeconomic assumptions, perhaps incorporating demographic change and economic growth. These projections typically have focused only on what changing weather patterns could mean for climate-relevant health outcomes. Although such an approach provides valuable insights, it also implicitly assumes the current drivers and distribution of vulnerabilities and capabilities, and the level of adaptation, will not change in the future. These are not plausible assumptions, so such projections may not provide realistic analyses to support policy- and decision-making. Different socioeconomic futures need to be considered to provide more credible analyses of the range of possible future health risks associated with climate change. Factors that could be considered include:

- possible advances in legislation, the built environment, technology, surveillance and control strategies, diagnosis and treatment, etc., and their deployment;
- progress in reaching sustainable development goals;
- level of education of women and policies that promote reproductive rights; and
- other factors that could alter future burdens of climate-related diseases and the status of the public health and health care infrastructure and institutions with which climate change could interact.

Projections of the possible impacts of climate change have primarily been based on scenarios described in the Intergovernmental Panel on Climate Change (IPCC) Special Report on Emission Scenarios (SRES) [1]. The SRES scenarios were developed to represent the range of driving forces (including demographics, economic growth, and technology development) and emissions in the scenario literature, including underlying uncertainties. The four main storylines (A1, A2, B1 and B2) of possible and internally consistent development pathways are described along demographic, social, economic, technological, and environmental dimensions. Each storyline projects resulting greenhouse gas and sulfur emissions and their evolution over this century. Several scenarios were developed for each storyline (total = 40) to examine the range of possible future emission pathways associated with similar assumptions about driving forces. By design, the SRES scenarios assumed no specific climate mitigation or adaptation policies and measures. Quantification of the storylines resulted in estimated emissions of greenhouse gases and sulfur that have been used by earth system models to project changes in temperature, precipitation, other weather variables, and sea level rise, over the course of this century. Impact modelers have used projected climatic changes to explore possible consequences of different scenarios for human and natural systems. Mitigation and adaptation researchers have used the scenarios to explore the possible effectiveness of policy options to avoid and manage projected risks.

The SRES scenarios are becoming dated in terms of scientific understanding and in their assumptions about demographic and socioeconomic change over time. For example, earth system models now include the full basket of greenhouse gases, more detailed projections are available for land use and land use change over the century, and demographic projections for mid- to late-century are quite different from those used in the SRES. New scenarios are needed to cover the wider range of greenhouse gas concentrations explored in the literature (including those that could be reached by mitigation measures) and to facilitate integration of mitigation, adaptation, and impact analyses. Policy- and decision-makers are asking not just about the magnitude and pattern of climate change and associated impacts, but also how alternative mitigation and adaptation polices could assist in managing projected risks and take advantage of possible opportunities. Answering these types of questions requires considering how variables not included in the SRES scenarios, such as inequality and governance, could evolve under different development pathways.

At the end of the SRES process, it was decided that the scientific community would lead further scenario development because scenarios developed by the research community have greater scientific credibility; the potential for much wider participation of research groups across disciplines and geographic regions; and the growing ability of the climate research communities involved to self-organize [2]. The IPCC has catalyzed the process by supporting some workshops (see below), but does not play organizing or supporting roles.

## 2. New Scenario Process

Extensive discussions of approaches to and the process for developing new scenarios started in 2006 and continued over many meetings and workshops [2,3]. Discussions involved members of the three main research communities working on aspects of climate science: earth system modeling; vulnerability, impacts, and adaptation (VIA; also called IAV) researchers; and integrated assessment modeling (IAM). In 2007, the Expert Meeting on Scenarios organized by the IPCC at Noordwijkerhout, Netherlands formalized a roadmap to develop new scenarios, following a three-step, so-called parallel process [4,5].

The parallel process describes a new approach to scenario development. Instead of the forward-looking process used for the SRES that starting with driving forces and their resulting emissions (from which atmospheric concentrations would be modeled), the scientific community agreed to first identify a small number of atmospheric concentrations of greenhouse gases (and their associated radiative forcing) in 2100, and then to simultaneously project climate change over the century and beyond, and to develop socioeconomic pathways to describe the evolution of elements over this century that could lead to the chosen concentrations [3,6].

The process being followed is a modification of the parallel process that is informed by an insight gained from the SRES [1] and confirmed by Van Vuuren *et al.* [7]: socioeconomic development pathways and greenhouse gas emissions are only loosely correlated. Instead of assuming that one development pathway would lead to a small range of atmospheric concentrations of greenhouse gases, it was realized that multiple demographic and socioeconomic development pathways can lead to any particular emission pathway, and any one socioeconomic pathway can lead to a range of emission pathways. For example, a relatively wealthy world with high population density could have high or low greenhouse gas emissions, depending on policies that encourage energy efficiency, development of low emission technologies, and other measures. This relative independence means that demographic and socioeconomic development pathways can be described separately from greenhouse gas emissions [2,8].

Based on this insight, the new scenario process includes:

- A preparatory phase designed to serve the needs of the earth system modeling community. Together with the IAM community, four Representative Concentration Pathways (RCPs) were chosen. The IAM community then determined the emissions that would produce each, taking into consideration emissions of greenhouse gases and short-lived species specified on a 0.5° latitude x 0.5° longitude grid, and including land use and land cover. The development of the four RCPs is documented in a special issue of *Climatic Change* [9]. The modelers made the smallest number of socioeconomic assumptions needed; the aim was not to provide the backstories on how emission pathways developed. Because the RCPs incorporate carbon dioxide and other greenhouse gases, they are described in terms of their radiative forcing in W/m^2^ in 2100 and their trajectory of change. The four RCPs are 2.6, 4.5, 6.0, and 8.5 W/m^2^, corresponding to carbon dioxide equivalent concentrations in 2100 of approximately 490 ppm, 650 ppm, 850 ppm, and 1,370 ppm, respectively. Note that RCP2.6 is a peak and decline pathway where radiative forcing peaks before 2100 and then declines (with negative emissions at the end of the century) to reach 2.6 W/m^2^ in 2100.
- A parallel phase involving the earth system modeling community and the wide range of research communities needed to develop socioeconomic scenarios. The new scenario development is in this phase. The RCPs are being used in simulations by earth system models as part of the Coupled Model Intercomparison Project (CMIP-5), producing projections of the magnitude and pattern of climate change over this century and, in some cases, to 2300 [10]. Projections from these experiments are assessed in the IPCC Fifth Assessment Report. The IAM and VIA communities are developing new descriptions of future socioeconomic conditions, the Shared Socioeconomic Pathways (SSPs). The process and architecture being used are described in another special issue of *Climatic Change* [2,8,11,12].
- An integration phase where scenarios for use in climate science research and assessment will be developed; this phase is just underway. These scenarios will integrate the socioeconomic development pathways with the climate change projections and with assumptions about climate mitigation and adaptation policies [11].

The new scenario process, particularly the development of the shared socioeconomic pathways, the shared climate policy assumptions, and the scenarios, is being coordinated by the International Committee on New Integrated Climate change assessment Scenarios (ICONICS; http://www.isp.ucar.edu/iconics) and the Integrated Assessment Modeling Consortium (IAMC).

## 3. Methods

Meetings of IAM and VIA researchers over the past two to three years facilitated progress on developing a framework for the socioeconomic development pathways ([13,14,15], and a workshop co-sponsored by the IPCC and the government of the Netherlands in the Hague in May 2012). Two proposed frameworks within which scenarios could be developed resulted from discussions at early meetings [7,16]. Ultimately, a unified framework was proposed: the scenario matrix architecture.

### 3.1. Scenario Matrix Architecture

Instead of providing packaged scenarios similar to those included in the SRES, the process is producing a toolkit of essential elements that researchers can use to tailor-make scenarios to address a wide range of policy- and decision-relevant questions. These elements are organized through a scenario matrix architecture, with qualitative and quantitative elements, from three sets of building blocks:

- Radiative forcing as described in the RCPs and resulting climate change;
- Socioeconomic development pathways; and
- Climate (mitigation and adaptation) policies.

In addition, marker (or reference) scenarios are under development to facilitate integration across disciplines and comparison within and across research communities [8].

The two main axes of the matrix are the level of radiative forcing (described in the RCPs); and a range of possible socioeconomic reference pathways (the SSPs, described in the next section) [8]. Scenarios are developed within the cells of the matrix—although not all cells will be populated (e.g., it is hard to imagine a scenario where the world is striving for sustainable development but with very high greenhouse gas emissions). When creating a scenario, additional assumptions may be needed about adaptation and mitigation policies to derive a scenario consistent with a given combination of a RCP and SSP; these additional assumptions are the Shared climate Policy Assumptions (SPAs, described later).

### 3.2. Shared Socioeconomic Pathways (SSPs)

Shared Socioeconomic Pathways define plausible alternative global pathways by which human and natural systems could evolve over the 21st century that can be “shared” across all or several RCPs (e.g., the same socioeconomic pathway can lead to multiple emission pathways), and include a narrative and a set of quantified measures [2,12]. The SSPs describe reference cases of alternative socioeconomic development pathways that would increase or decrease challenges to adaptation or mitigation regardless of climate change outcomes or policies, e.g., they do not assume new climate policies and assume no significant climate feedbacks on development [1,11]. Although these assumptions may be unrealistic, they are necessary because the outcomes of scenario-based research and assessment will include climate change projections, impacts, and climate policy responses. Because these outcomes are the subject of study, they cannot be built into the pathways describing radiative forcing (RCPs) or socioeconomic development (SSPs). It is important to note that some adjustments may be needed of a SSP narrative and/or quantification when it is combined with a RCP to create a scenario, so that the SSP remains within the appropriate challenges space (described in further detail below).

To encompass a wider range of possible development pathways of relevance for climate change research than were represented in the SRES, it was decided to define five SSPs along axes describing increasing socioeconomic and environmental challenges to adaptation and to mitigation effectiveness [12]. The SSPs include the socioeconomic factors that create the conditions determining the ease or difficulty of adaptation and mitigation, such as demographic, political, social, cultural, institutional, lifestyle, economic, and technological variables, and their trends. Also considered are conditions of ecosystems and ecosystem services affected by human activities, such as air and water quality, and biodiversity. This approach facilitates exploration of the possible impacts associated with mitigating to a certain level of radiative forcing, and of the extent of efforts required to adapt to that level.

Defining the SSPs by challenges to adaptation and mitigation is very different from the SRES and the Millennium Ecosystem Assessment scenarios that defined their axes by key socioeconomic driving forces assumed to be principal uncertainties determining the outcomes of interest [1,17]. Instead, the SSPs use the outcomes of interest to define the axes [12]. The axes and the space they create are not intended to indicate which combination of socioeconomic elements would produce a given set of challenges, nor which elements are the key uncertainties [12].

Challenges to mitigation include factors and trends that generate high reference emissions in the absence of climate policy and reduce the social capacity to mitigate those emissions [12]. These include inadequate national and international policy-making institutions, insufficient investment in research and development, lack of (or lack of access to) viable technologies, and insufficient financial and other resources to support effective mitigation, such as political will and human and social capital [12,18,19]. High reference emissions could result from many combinations of high population growth rates, rapid conventional economic growth, energy-intensive economies, carbon-intensive energy use, and the like. Not all factors need operate in the same direction to result in high (or low) emissions.

Socioeconomic challenges to adaptation increase the risks associated with any given level of climate change by making adaptation more difficult [12]. Challenges to adaptation include factors such as poverty and the distribution of wealth, less effective national and international organizations and institutions, water and food insecurity, limited access to education, and high levels of unplanned urbanization.

**Figure 1:**
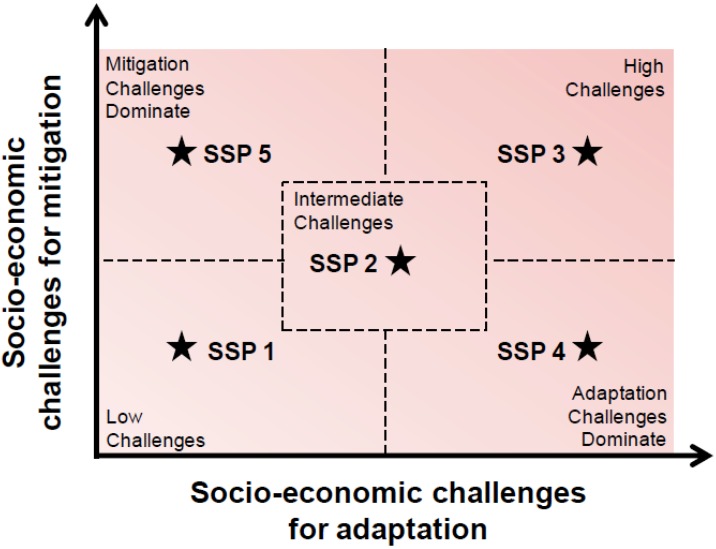
Challenges space for shared socioeconomic pathways.

Source: [12].

The challenges space is divided into five domains so that there is a middle development pathway (Figure 1). Along the diagonal axis are (1) sustainability (low challenges to adaptation and mitigation); (2) middle-of-the-road (medium challenges); and (3) fragmentation (high challenges). Off axis are (4) inequality (low challenges to mitigation and high challenges to adaptation); and (5) conventional development (high challenges to mitigation and low challenges to adaptation). The narratives and quantifications of the SSPs describe patterns and trends of how these worlds evolve over time, relative to the middle-of-the-road pathway, which itself evolves. Again, the SSPs do not include assumptions about new adaptation and mitigation polices.

Key characteristics of the SSPs include [2,12]:

- A focus on global socioeconomic and environmental trends and large world regions over the 21st century;
- Qualitative narratives and quantifications sufficient to distinguish the five domains;
- Information typically used as inputs to integrated assessment models of the global energy-economy-land use system or to global scale impacts models, such as assumptions about future demographics, economic development, and degree of global integration. Typical outcomes of these models are not included.
- Sufficient information to create the boundary conditions for extending the SSPs to develop scenarios at regional and sectoral scales.

Based on these characteristics, elements incorporated or being explored for incorporation into the SSPs include [12,20,21]:

- Demographics, including population and age structure, and urban vs. rural populations;
- Economic development, including global and regional gross domestic product, trends in productivity, and proportion of population in extreme poverty;
- Welfare (including human development), educational attainment, and health;
- Environmental and ecological factors, including air, water, and soil quality;
- Resources, including fossil fuels and renewable energy potentials;
- Institutions and governance, including existence, type, and effectiveness;
- Technological development, including type and diffusion of technological progress;
- Broader societal factors, including attitudes to the environment and sustainability, and life styles; and
- Non-climate policies, such as policies on development, technology, urban planning, transportation, energy, and environment.

### 3.3. Shared Climate Policy Assumptions (SPAs)

Scenarios designed to achieve a particular RCP may need to include mitigation and adaptation policies to reduce emissions to the specified level of radiative forcing and to have the designated capacity to cope with resulting climate change [11]. Given the plethora of possible adaptation and mitigation policies, Shared climate Policy Assumptions (SPAs) will describe sets of policies that can be used in impacts, adaptation, and mitigation studies to investigate the consequences of particular policy approaches. The intent is for SPAs to capture key climate policy dimensions not specified in the SSPs, describing features such as global and sectoral coverage of greenhouse gas reduction regimes and/or adaptation effectiveness.

## 4. Results and Discussion

### 4.1. Health Trends of Relevance to Scenarios

Of the wide variety of health outcomes that could be affected by climate change, the ones with the greatest worldwide burden are undernutrition, malaria, and diarrheal disease [22]. These also are among the main causes of preventable childhood mortality. The Global Burden of Disease study 2010 estimated that of the 7.6 million children who died in the first 5 years of their life in 2010, 64.0% (4.9 million) died of infectious causes, with pneumonia, diarrhea, and malaria the leading causes of death [23]. Diarrhea caused 10.5% of all childhood deaths (0.5 to 1.2 million), and malaria caused 7.4% (0.4 to 0.7 million). These numbers declined over recent years. For example, the burdens of malaria and diarrheal disease in children 1–59 months declined 4.0% per year between 2004 and 2010 for malaria, and between 2000 and 2010 for diarrheal disease.

Some of this progress may be due to efforts to achieve the Millennium Development Goals (MDGs), which include targets that helped catalyze major national and international efforts to reduce the burdens of undernutrition (MDG 1.C), malaria (MDG 6.C), and diarrheal disease (part of MDG 4). Significant progress on some targets had been made as of June 2013, measured against a 1990 baseline [24].

Target 1.C: *Halve, between 1990 and 2015, the proportion of people who suffer from hunger*, has been achieved in northern Africa, eastern and south-eastern Asia, Latin America and the Caribbean, and Caucasus and Central Asia [25]. Although progress has been made, there is a very high prevalence of hunger in Sub-Saharan Africa, and of high hunger in southern Asia. There has been no progress or deterioration in western Asia and Oceania. In 2011–2013, 842 million people (approximately 12% of the world population) were estimated to suffer from chronic hunger [25]. The total number of undernourished people decreased 17% since 1990-1992. It should be kept in mind that these are national estimates of the prevalence of undernourishment that summarize over considerable sub-national diversity.

Target 4.A: *Reduce by two-thirds, between 1990 and 2015, the under-five mortality rate*, has been achieved in northern Africa, eastern, south-eastern, and western Asia, and Latin American and the Caribbean [24]. Although progress has been made, high mortality remains in Sub-Saharan Africa, and moderate mortality in southern Asia, Oceania, and Caucasus and central Asia.

Target 6.C: *Have halted by 2015 and begun to reverse the incidence of malaria and other major diseases*. On average, malaria continues to kill a child every minute [26]. Just under half of the world population is at risk of contracting malaria. An estimated 219 million cases occurred in 2010, causing approximately 660,000 deaths, most of them children under five in Africa. Progress in reducing malaria cases and mortality has been faster in countries with lower prevalence.

These trends are part of the backdrop against which narratives and quantifications of possible socioeconomic futures are being developed.

### 4.2. Health in the SSPs

A brief summary of each SSP is followed by interpretations of that SSP for public health and health care. These summaries are based on the initial sketches of the SSPs in [15]. A paper with full descriptions of the SSP narratives is in preparation. The working titles of the SSPs are provided.

*Sustainability* (SSP1) is a world with low challenges to adaptation and mitigation. It is a world making relatively good progress towards sustainability with global cooperation, facilitated through effective international organizations and institutions, on achieving development goals and reducing resource intensity and fossil fuel dependency. Low-income countries rapidly develop, with fewer people below the poverty line, reductions in inequality within and across countries, higher rates of female education, slower population growth, improved population health, increases in planned urbanization, rapid development of clean energy technologies, and a high level of awareness regarding environmental degradation.

SSP1 depicts a world where population health improves significantly, with increased emphasis on enhancing public health and health care functions that, in turn, increase the capacity to prepare for, respond to, cope with, and recover from climate-related health risks. Coordinated, worldwide efforts through international institutions and non-governmental organizations to achieve sustainable development goals increase access to safe water, improved sanitation, medical care, education, and other factors in underserved populations. Social capital increases, resulting in more effective community-based efforts to manage local health and environmental quality. These improvements reduce the burden of these health outcomes before considering any impacts of climate change. Life expectancies increase in low-income countries with decreasing burdens of key causes of childhood mortality (undernutrition, diarrheal diseases, and malaria). However, as more children survive to adulthood, burdens of non-communicable diseases increase, although changes in dietary patterns and reductions in air pollution from burning fewer fossil fuels lower the burden of some chronic diseases. Funding increases for public health and health care organizations and institutions to improve (1) monitoring and surveillance systems of climate-related health outcomes that integrate health and environmental information, and of adaptation policies and measures; (2) research on and modeling of the health risks of climate change; (3) use of iterative management approaches to increase the effectiveness of adaptation policies and measures; (4) the number of health care and public health professionals and practitioners trained in climate change risks; and (5) technology development and deployment, such as identifying new diagnosis and treatment options and increasing use of telemedicine. Enhanced cooperation across sectors minimizes co-harms from adaptation options implemented in other sectors, such as agriculture and water. This cooperation and coordination leads to integrated effects to address food and water security, and to enhance protection from extreme weather and climate events.

*Middle of the road* (SSP2) is a world with medium challenges to adaptation and mitigation. It is a world where trends typical of recent decades continue, with slow progress towards achieving development goals, reductions in resource and energy intensity at historic rates, and slowly decreasing fossil fuel dependency. There is a moderate level of international cooperation and technology investments. The limited number of comparatively weak global institutions lead to uneven development of low-income countries, with some countries making relatively good progress and others left behind. Urbanization follows a similar pattern. Educational investments are not high enough to rapidly slow population growth, particularly in low-income countries. Most economies are politically stable, with partially functioning and globally connected markets.

SSP2 depicts a world where population health improves, although not as quickly as in a sustainability world. Progress in reducing the burden of climate-related health outcomes in low-income countries is slow and uneven, with not all low-income countries making progress. Public health and health care international institutions and nongovernmental organizations have an inadequate and not well-coordinated focus on addressing the burden of climate-related health outcomes. Access to safe water, improved sanitation, and medical care slowly improve. For many low-income countries, the burdens of infectious and chronic diseases increase (e.g., the double burden of disease), thus continuing the disproportional impacts of climate-relevant health outcomes on children. Multiple factors contribute to some countries making slower progress in reducing health burdens, including, in some low-income countries, high burdens of climate-related diseases combined with moderate to high population growth. Other contributing factors are constraints to adaptation because of limited human and financial resources and personnel, weak surveillance and control programs, and insufficient access to new technologies and expertise. Funding for public health infrastructure and health care falls below requirements, with inadequate resources and international commitment for (1) integrated monitoring and surveillance systems; (2) research on and modeling of the health risks of climate change; (3) iterative management approaches; (4) training health care and public health professionals and practitioners; and (5) technology development and deployment. Limited cooperation across sectors increases the probability of co-harms from adaptation and mitigation options implemented in other sectors. This lack of integration increases the health risks of food and water insecurity, and limits progress on managing the risks of extreme events and disasters. Adverse health outcomes associated with the burning of fossil fuels increase (with moderate progress on regulations on end-of-pipe measures to reduce air pollutants), particularly in rapidly industrializing economies, leading to increasing burdens of associated chronic diseases.

*Fragmentation* (SSP3) is a world with high challenges to adaptation and mitigation. It is a world separated into regional blocks with little coordination between them. This world is failing to achieve global development goals, with little progress in reducing resource intensity and fossil fuel dependency. The regions are characterized by extreme poverty and pockets of moderate wealth, with the bulk of countries struggling to maintain living standards for their strongly growing populations. Mortality rates are high, with many children dying from preventable diseases, including undernutrition, diarrheal disease, and malaria. Countries focus on achieving regional energy and food security goals with little international cooperation and low investments in technology development and education. Most urban growth in low-income countries is in unplanned settlements. Governance and institutions are weak, with a lack of collaboration and consensus.

SSP3 depicts a world characterized by increasing mortality from climate-related health outcomes (particularly undernutrition, diarrheal diseases, and malaria) and possibly falling life expectancy in low-income countries because of increased childhood mortality, although some sub-regions enjoy better health. Developed and developing countries experience a double burden of infectious and chronic climate-related health outcomes. Challenges to improving human wellbeing in low- and middle-income countries include weak international and regional institutions, limited international cooperation, low investments in public health and health care infrastructure, and too few public health and health care personnel to address needs. Climate-related infectious diseases increase in developed countries with reduced funding for surveillance and monitoring programs, limited investment in research and technology development, less effective institutions for ensuring food and water safety, and poor access to health care. Urbanization mostly fails to improve access to safe water and improved sanitation, thus increasing the burden of infectious diseases, particularly in children and those living in unplanned settlements. Increasing reliance on fossil fuels and limited progress on regulations on end-of-pipe measures to reduce air pollutants, particularly in low- and middle-income countries, increases the burden of respiratory and cardiovascular diseases. There is very high vulnerability to the impacts of extreme weather and climate events. To the extent to which regions focus on health issues, they do so only for issues of concern within the region; wealthier regions do not invest in research and development to help less well off regions manage health risks. Limited coordination also occurs within countries, resulting in adaptation and mitigation actions in other sectors sometimes adversely affecting health. Large regions of the world are food and water insecure, with limited trade to move food across regions.

*Inequality* (SSP4) is a world with low challenges to mitigation and high challenges to adaptation. It is a highly unequal world within and across countries, with regular social conflict and unrest. A relatively small, rich global elite is responsible for much of the emissions, which they can mitigate at low cost, while a larger, poorer group contribute little to emissions and are highly vulnerable to the impacts of climate change. Rural areas and mega-cities house a large fraction of relatively poor and less educated people, who lack the capacity to protect themselves from extreme weather events. Access to high quality education, health services, and family planning is limited, leading to high population growth in low-income countries. Economic uncertainty in industrialized countries results in relatively low fertility and low population growth. Global energy corporations invest in research and development to hedge against potential resource scarcity or climate policy, developing (and applying) low-cost alternative technologies.

SSP4 depicts a world where health differs from worlds with high challenges to adaptation and mitigation in the magnitude and extent of greenhouse gas emissions. Low challenges to mitigation make some aspects of this world similar to worlds with low challenges to adaptation and mitigation, with lower burdens of some chronic diseases from changes in dietary patterns and reductions in air pollution from less reliance on fossil fuels. This coexists with features of fragmented worlds (SSP3), with mortality increasing from other climate-related health outcomes (particularly undernutrition, diarrheal diseases, and malaria) in low- and middle-income countries with lower investment in public health and health care research, development, and training, poor access to health care, less effective institutions for monitoring water and food security, and high levels of unplanned urbanization. International and regional institutions are weak, providing limited funding and technical expertise for reducing health burdens in low-income countries who don’t have the human or financial resources, or access to technology, to do so themselves. This is a highly vulnerable world, with limited capacity to avoid, prepare for, and cope with outbreaks of infectious diseases, episodes of high food insecurity, and extreme events. Limited co-operation across sectors increases co-harms from adaptation and mitigation options implemented in other sectors.

*Conventional development* (SSP5) is a world with low challenges to adaptation and high challenges to mitigation. It is a world focusing on self-interested conventional development oriented toward economic growth as the solution to social and economic problems. The preference for rapid conventional development leads to an energy system continuing to be dominated by fossil fuels, resulting in high emissions. There is a strong push for developing countries to follow the fossil and resource-intensive development model of industrialized countries, focusing on economic growth aided by consumerism and resource intensive status consumptions. This world attains human development goals, with robust economic growth, rapid urbanization, highly engineered infrastructure, and highly managed ecosystems. There also are strong investments in health, education, and institutions to enhance human and social capital. There is faith in the ability to manage social and ecological systems, and with relatively little specific proactive efforts to avoid potential global environmental impacts.

SSP5 depicts a world where health improves significantly, but not as much as in a sustainability world with low challenges to adaptation and mitigation (SSP1) because energy intensive systems increase the burning of fossil fuels, although progress on regulations on end-of-pipe measures reduces some air pollutants. As in SSP1, social capital increases. Because the challenges for local management of environmental quality are larger, the burden of chronic diseases is somewhat higher than in SSP1. Human and financial resources increase for public health and health care organizations, with a priority for improving the health of populations in low-income countries. The effectiveness of international organizations, and the emphasis on international and regional cooperation, enhances the capacity of health care and public health institutions to avoid, prepare for, and cope with climate-related health risks in underserved populations. This increased capacity, along with research and technology development and deployment, facilitates significant progress in achieving sustainable development goals, except those directed at energy efficiency, through improving access to safe water, improved sanitation, medical care, education, and other factors that reduce vulnerability. In addition, highly engineered urbanization has positive health effects by increasing access to safe water and improved sanitation, reducing the burden of water- and foodborne diseases. Therefore, life expectancies increase in low-income countries through decreasing burdens of undernutrition, diarrheal diseases, and malaria. At the same time, the burden of chronic diseases increases with more children surviving to adulthood. There is enhanced cooperation across sectors, limiting the potential for co-harms from adaptation and mitigation options.

## 5. Conclusions

The new scenario process offers a significant opportunity for the health sector to develop scenarios relevant for questions being asked and decisions that need to be taken by policy- and decision-makers about:

- the extent to which climate change could affect the geographic range, seasonality, and incidence of climate-related health outcomes under different assumptions of future socioeconomic development;
- the extent to which adaptation and mitigation policies could avoid those health risks and increase the capacity of the health sector to prepare for, cope with, and recover from climate change, and what these policies will cost; and
- how the balance of adaptation and mitigation policies could alter health burdens over time.

The toolkit being produced focuses on the global scale, providing narratives and quantifications that create boundary conditions for sectoral and regional extensions. Creating health-specific extensions is the responsibility of the health sector.

Scenarios developed from this process allow exploration of the interplay between development pathways and climate change, furthering understanding of where best to place scarce human and financial resources to manage risks. For example, climate change alone is projected to increase mortality from heatwaves [27]. Scenarios incorporating SSPs and RCPs can be used to explore the extent to which different patterns of urbanization, population settlement, demographics, and climate change could make it easier or more difficult to prepare for and effectively manage heatwave risks. Understanding of these patterns can be used to guide and advocate for more robust development choices, including mitigation to reduce climate change risks later in the century.

The health sector will need to self-organize to produce their own SSP extensions to underlie projections of health impacts under different degrees of climate change and patterns of development. However, the sector is not currently organized to do so. Among the consequences of the IPCC leading previous scenario development is that there is no tradition of international and national organizations funding health scenario development. To facilitate the process of developing health extensions, it would be helpful for some international and national public health and health care organizations to sponsor an international meeting to discuss and agree which elements would be most useful to add to the SSPs to facilitate understanding of and managing possible future health risks. Such a meeting should include the representatives of relevant communities, including climate change and health impacts, mitigation and adaptation, and development. For example, understanding how climate change could affect the future geographic range of infectious diseases and malnutrition in low-income countries would ideally include projections of how maternal and childhood mortality could change independent of climate change under different development pathways. These and other potentially desirable variables are important measures in their own right as well as indicators of the ability of nations (and the non-governmental and other organizations operating in a particular country) to deliver primary health care and public health services. The limited efforts to project how socioeconomic and other key determinants of health burdens could evolve over coming decades could be augmented to provide a broader range of projections of socioeconomic and health-determining variables over longer temporal scales and at finer spatial resolution. It may be helpful to create partnerships with individuals and groups working on issues such as the global burden of disease and measures of health governance. The proposed meeting also could discuss the process for constructing and vetting the extended narratives, how to produce the desired quantifications, the process for coordinating these activities, and possible funding opportunities.

Another issue that would be useful to consider is creating scenarios over shorter time periods than this century. Few health adaptation decisions will be affected by the magnitude and pattern of climate change later in the century. Assuming the health sector practices iterative risk management, then adaptation decisions, such as modifications of surveillance and monitoring programs to take changing weather patterns into account, can focus on projected climate change over the next few decades. Infrastructure is one exception; decisions on the placement and character of infrastructure will be more robust if the risks later in the century, including uncertainties, are considered.

Richer narratives and quantifications of the SSPs would be helpful for these shorter-term decisions. One approach is to use the extensive data gathered on the progress towards meeting the MDGs and estimates of when (and where) particular targets will be achieved. One could view the middle-of-the-road SSP (SSP2) as a world that achieves the MDGs perhaps a bit more slowly than is the current situation. The sustainability SSP (SSP1) is a world moving more quickly to achieve all MDGs and the sustainability goals (SDGs) that will succeed them. The fragmented SSP (SSP3) is a world that achieves the MDGs, if at all, only after a long delay. Each SSP could be enriched with more narrative elements and information on how the MDGs and SDGs could evolve over the next two to three decades. In addition, because poverty is a key determinant of vulnerability, it also could be useful to explore the possibility of constructing multidimensional poverty indexes to describe how vulnerability could evolve under different socioeconomic development pathways (e.g., [28]). Other aspects to be considered include variables related to the basic material needs for a good life, good social relations, security, and freedom of choice and action [29].

The possibilities for SSP elaborations are large, including over a range of spatial and temporal scales. The questions are what scenarios would be most useful to health decision- and policy-makers to support their efforts to increase resilience to the health risks of climate variability and change under different development pathways, and how to go about developing them.
